# Frequency-domain CBE imaging for ultrasound localization of the HIFU focal spot: a feasibility study

**DOI:** 10.1038/s41598-020-62363-7

**Published:** 2020-03-25

**Authors:** Kun Yang, Qiang Li, Hao-Li Liu, Chin-Kuo Chen, Cheng-Wei Huang, Jheng-Ru Chen, Yu-Wei Tsai, Zhuhuang Zhou, Po-Hsiang Tsui

**Affiliations:** 1 School of Microelectronics, Tianjin University, Tianjin, China; 2 Department of Electrical Engineering, Chang-Gung University, Taoyuan, Taiwan; 3 Department of Otolaryngology – Head and Neck Surgery, Chang Gung Memorial Hospital and Chang Gung University, Taoyuan, Taiwan; 4 Department of Medical Imaging and Radiological Sciences, College of Medicine, Chang Gung University, Taoyuan, Taiwan; 5 College of Life Science and Bioengineering, Beijing University of Technology, Beijing, China; 6 Medical Imaging Research Center, Institute for Radiological Research, Chang Gung University and Chang Gung Memorial Hospital at Linkou, Taoyuan, Taiwan; 7 Department of Medical Imaging and Intervention, Chang Gung Memorial Hospital at Linkou, Taoyuan, Taiwan

**Keywords:** Ultrasonography, Biomedical engineering

## Abstract

High-intensity focused ultrasound (HIFU) is a well-accepted tool for noninvasive thermal therapy. To control the quality of HIFU treatment, the focal spot generated in tissues must be localized. Ultrasound imaging can monitor heated regions; in particular, the change in backscattered energy (CBE) allows parametric imaging to visualize thermal information in the tissue. Conventional CBE imaging constructed in the spatial domain may be easily affected by noises when the HIFU focal spot is visualized. This study proposes frequency-domain CBE imaging to improve noise tolerance and image contrast in HIFU focal spot monitoring. Phantom experiments were performed in a temperature-controlled environment. HIFU of 2.12 MHz was applied to the phantoms, during which a clinical scanner equipped with a 3-MHz convex array transducer was used to collect raw image data consisting of backscattered signals for B-mode, spatial-, and frequency-domain CBE imaging. Concurrently, temperature changes were measured at the focal spot using a thermocouple for comparison with CBE values by calculating the correlation coefficient *r*. To further analyze CBE image contrast levels, a contrast factor was introduced, and an independent *t*-test was performed to calculate the probability value *p*. Experimental results showed that frequency-domain CBE imaging performed well in thermal distribution visualization, enabling quantitative detection of temperature changes. The CBE value calculated in the frequency domain also correlated strongly with that obtained using the conventional spatial-domain approach (*r* = 0.97). In particular, compared with the image obtained through the conventional method, the contrast of the CBE image obtained using the method based on frequency-domain analysis increased by 2.5-fold (4 dB; *p* < 0.05). Frequency-domain computations may constitute a new strategy when ultrasound CBE imaging is used to localize the focal spot in HIFU treatment planning.

## Introduction

High-intensity focused ultrasound (HIFU) is a therapeutic technique for inducing thermal lesions in tissues by focusing acoustic energy to generate a high temperature at the focal point. HIFU has been increasingly explored as a noninvasive thermal treatment for ablating tumors. HIFU treatment has been established to play a key role in shortening postoperative recovery time and reducing the risk of complications^1,2^.

During HIFU treatment, correct sound beam distribution is essential to avoid thermal damage to surrounding normal tissues. However, phase aberrations caused by tissue inhomogeneities typically make the practical heating point shift from the geometric focus^3^. Therefore, targeting the focal spot is a critical step in HIFU treatment planning. Currently, magnetic resonance (MR) imaging and ultrasound imaging are two major modalities for the localization of the HIFU focal spot in treatment planning^4^. MR imaging is a reliable method for localizing the HIFU focal spot because it allows detection of the focal spot with a temperature change of 1 °C^5,6^. However, MR imaging is expensive with limited availability, and its device design and operating environment must meet nonferromagnetic requirements for materials and components. By contrast, ultrasound imaging has several advantages in terms of cost-effectiveness, real-time capability, and low compatibility requirements. In particular, the temperature increase alters the properties of ultrasound backscattered signals, including the echo time shift (caused by changes in the tissue thermal expansion and speed of sound)^7–9^, acoustic attenuation^10^, and change in backscattered energy (CBE)^11–13^. This implies that ultrasound parameters may serve as an alternative for localizing the HIFU-induced focal spot.

However, not every ultrasound parameter can be effectively used to monitor the focal spot for HIFU treatment planning. During the planning stage, a small temperature increment is typically generated at the focal spot to avoid heat deposition in the target^14^. The acoustic attenuation coefficient is unsuitable for monitoring the focal spot because it exhibits significant changes only at temperatures exceeding 50 °C^10^. The echo time shift (or thermal strain) increases with temperatures between 37 and 50 °C in tissues^4,9,15^, and it has been applied to the development of systems and algorithms for monitoring HIFU^16–18^. However, some factors (e.g., thermal expansion, different intensity levels, and variations in speed of sound for different tissue types) affect estimations of the echo time shift^19^. Thermal strain can also be mixed up with mechanical strain if mechanical deformations are present^20^. Although high-frame-rate imaging has been proposed to address the problem of *in vivo* tissue motion^21^, sufficient system resources are required to support imaging and computations.

Compared with the echo time shift, the CBE may be more viable for ultrasound localization of the HIFU focal spot. The CBE is caused primarily by thermal dependencies of the backscatter coefficients for scatterers; it varies almost monotonically with temperatures from 37 to 50 °C^11^. Conventionally, a CBE image is constructed by pixel-to-pixel calculation of the ratio of the backscattered energy between each temperature and the reference temperature^13^. Conventional CBE imaging is sensitive to changes in the signal features of backscattering and speckle motion; however, tracking and compensation of speckle motion may be ignored if CBE imaging is used only for visualizing temperature changes without considering the estimation accuracy for CBE^22^. After that, CBE imaging was further applied to monitoring HIFU, demonstrating the potential of CBE in HIFU focal spot localization^21,23^; whereas, CBE imaging is accompanied by tail-shaped artifacts occurring below the focal spot when monitoring HIFU, degrading the image contrast to some degree^23^. Thus, considering the presence and unavoidability of CBE artifacts, we must strive to enhance CBE imaging contrast to benefit the localization of the HIFU focal spot without sacrificing accuracy in representing temperature change.

Conventionally, algorithms for ultrasound CBE imaging are designed in the spatial domain^24,25^. In general, a major drawback for spatial-domain CBE imaging is that the CBE computation is easily affected by noises, particularly when the CBE is measured in decibels (dB) to describe the intensity ratio on a logarithmic scale. During HIFU treatment, noise sources include power electrical facilities and acoustic interferences, which produce unfavorable noises for ultrasound CBE imaging. Alternatively, estimating the CBE through a spectral comparison in the frequency domain may be a feasible strategy. Through a frequency-domain transformation, a frequency selection can be performed for data analysis to provide improved noise tolerance and image contrast for CBE imaging during HIFU focal spot monitoring.

This study proposes an algorithmic scheme for frequency-domain CBE imaging to localize the HIFU focal spot. *In vitro* phantom experiments were conducted to validate the proposed method. Compared with conventional CBE maps, frequency-domain CBE imaging provided an improved contrast for HIFU monitoring; the CBE value also preserved a high correlation with temperature changes.

## Materials and methods

### Experimental setup

**Figure 1:**
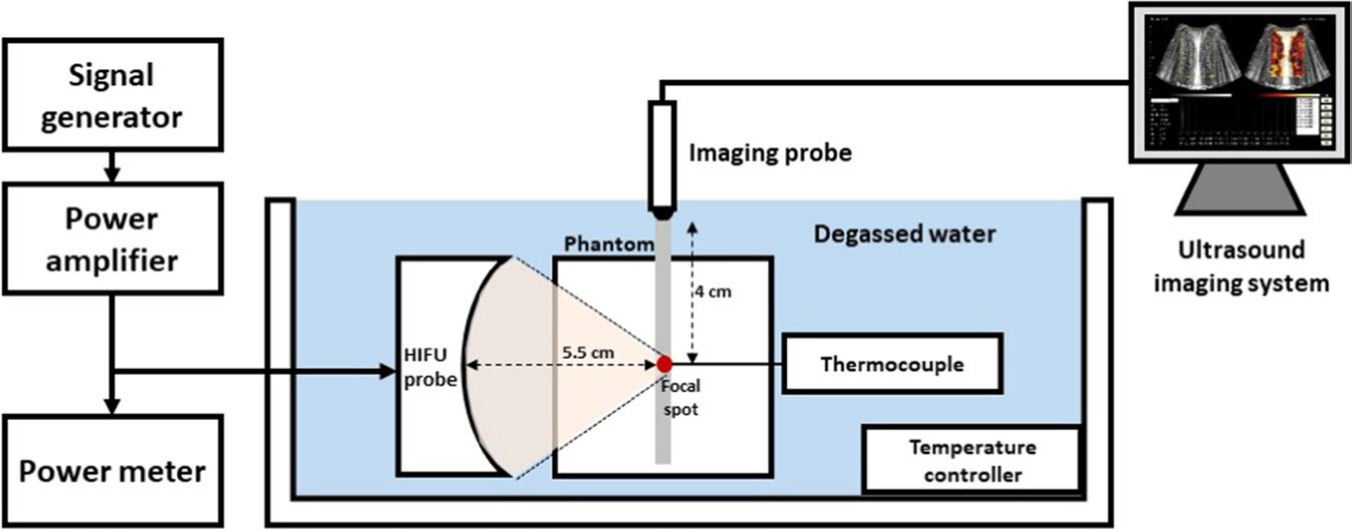
Experimental setup involved a self-assembled HIFU system, a clinical ultrasound system, and a thermocouple. The HIFU system comprised a function generator, a gain-variable power amplifier, a power meter, and a HIFU probe. The HIFU and imaging focal spots were at the same location.

The experimental system setup is illustrated in Fig. 1 and included a self-assembled HIFU system, a clinical ultrasound system (Model T3000, Terason Inc., Northborough, MA, USA), and a thermocouple (TM-947SD, Lutron Inc., Taipei, Taiwan). The HIFU system comprised a function generator (AFG 3022B, Tektronix Inc., Beaverton, OR, USA), a gain-variable power amplifier (150A100B, Amplifier Research, Souderton, PA, USA), a power meter (Model 4421, Bird Inc., Solon, OH, USA), and a HIFU probe (Sonic Concepts Inc., Bothell, WA, USA). The central frequency and focal length of the HIFU probe were 2.12 MHz (bandwidth: 1.96–2.28 MHz) and 5.5 cm, respectively. To generate the HIFU beam, the function generator was used to output a 2.12-MHz continuous sinusoidal wave, which was then amplified by the power amplifier for driving the HIFU probe. The driving power was monitored by the power meter. The clinical ultrasound system equipped with a convex array transducer (Model 5C2, Terason Inc., Northborough, MA, USA) was used for collecting ultrasound backscattered radiofrequency (RF) signals to monitor HIFU procedure. The imaging transducer frequency and the pulse length were 3 MHz (bandwidth: 2.11–3.69 MHz) and 2.3 mm, respectively. The thermocouple inserted into the phantom was responsible for measuring the temperature at the HIFU focal spot. The phantom was placed in an acrylic case, and a positioning hole was created on the wall of the case to facilitate the insertion of the thermocouple into the position where the imaging probe was confocal with the HIFU probe. The environmental temperature in the water tank, where the phantom was placed, was controlled by a temperature controller (I-629, ISTA Tech., Taoyuan, Taiwan) at approximately 33 °C.

### Experimental procedures and data acquisition

Phantom experiments were performed to evaluate the performance of conventional spatial-domain CBE imaging and of the proposed frequency-domain CBE imaging in HIFU focal spot localization. The phantoms were prepared by adding 10 g of graphite powder (acoustic scatterers) with a diameter of <20 μm (Model 282863, Sigma-Aldrich, St. Louis, MO, USA) into agar solution produced by dissolving 2 g of agar powder in 200 mL of water. During the cooling period, a stirrer was used to suspend graphite powder in the agar solution until the solution was solidified. According to a previous study^26^, the applied ratio between the weight of graphite powder and the volume of the solution ensures the appearance of fully developed speckles in ultrasound B-mode images.

**Figure 2:**
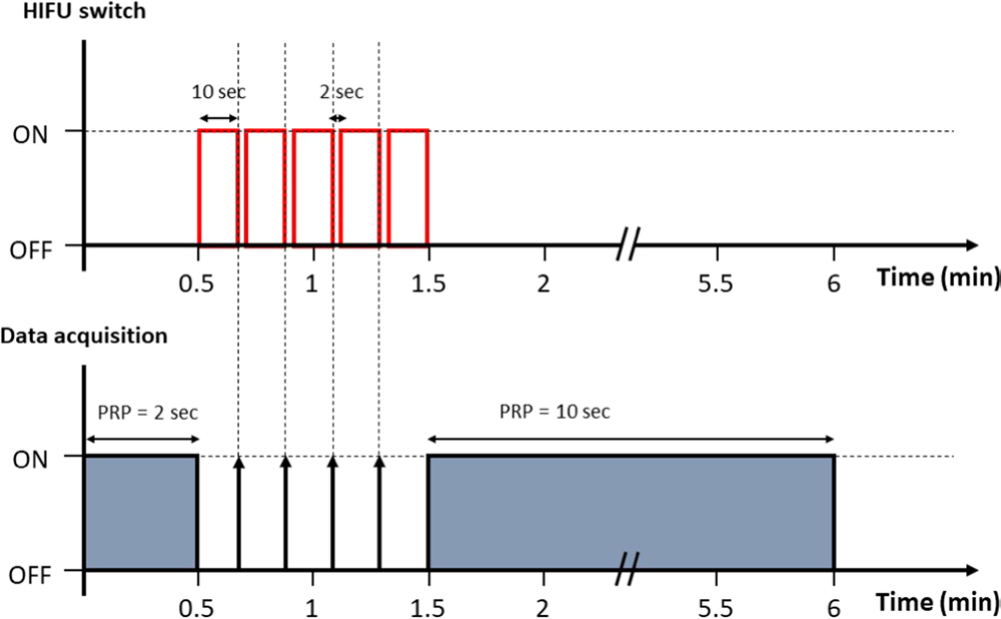
Sequence control for HIFU transmission and data acquisition, including the standby, heating, and cooling stages. To avoid the effect of the HIFU beam on ultrasound imaging, raw image data were acquired in each HIFU-off interval.

To simulate different thermal doses (temperature changes) at the HIFU focal spot, the driving voltages were set at 130, 170, and 210 mV (peak-to-peak value), corresponding to the output power levels of 5, 10, and 15 W, respectively. A phantom was placed in the water tank, and the thermocouple was inserted into the phantom for temperature monitoring. The imaging transducer was also immersed in the water and positioned above the phantom. Prior to the experiments, additional adjustments and calibrations were necessary for the experimental setup to allow both the thermocouple and the HIFU focal spot to be confocal and fall into the imaging plane. The experiments were initiated when the initial temperature of the phantom reached 33 °C. The sequence control for HIFU transmission and data acquisition can be divided into three stages, as presented in Fig. 2. The first stage is defined as the standby period lasting 30 s. In this stage, the HIFU system was off, and raw image data were acquired using the ultrasound imaging system every 2 s. The first frame of the raw data was used as the reference for the analysis of CBE imaging. The HIFU system was turned on in the second stage, from the 31^st^ to the 90^th^ s, which comprised five 10-s heating phases with 2-s HIFU-off intervals between each heating phase. To avoid the effect of the HIFU beam on ultrasound imaging, the raw image data were acquired in each HIFU-off interval. In the third stage, from the 91^st^ to the 360^th^ s, the HIFU system was turned off for cooling the phantom; during this period, the raw image data were acquired every 10 s. During HIFU procedure, the temperature data measured by the thermocouple were also collected.

The raw image data consisted of 128 A-lines of backscattered RF signals at a sampling rate of 12 MHz. The imaging depth and focal length were approximately 8 and 4 cm, respectively. For each output power, a total of five independent phantoms were used to repeat measurements. The acquired ultrasound RF signals were further processed for B-mode and CBE imaging. The details of the algorithms are provided as below.

### Ultrasound B-mode, spatial- and frequency-domain CBE imaging

**Figure 3:**
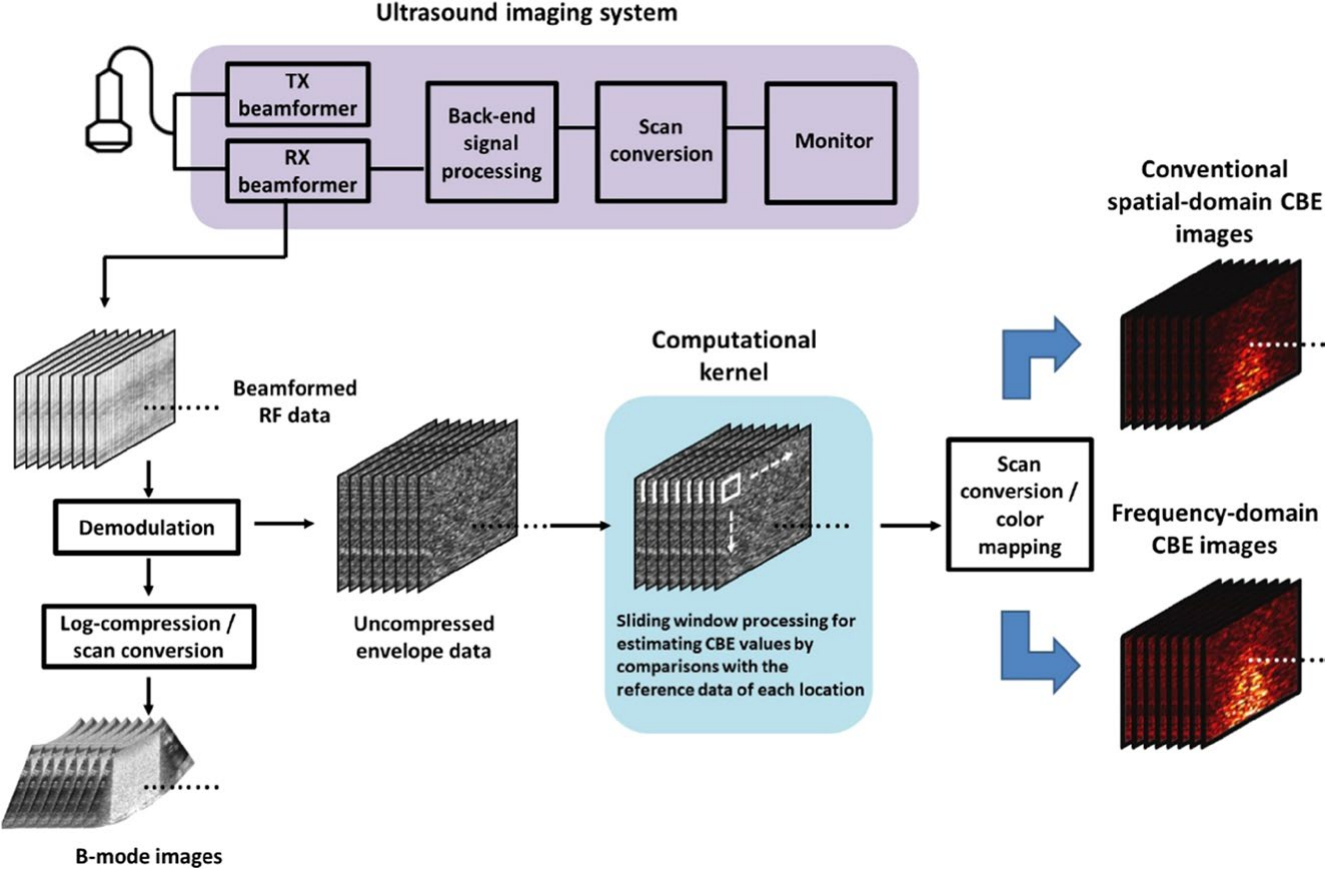
Algorithmic scheme for ultrasound B-mode, spatial-, and frequency-domain CBE imaging. The window-to-window computational scheme was used because it supports the acquisition of local ultrasound signals with finite lengths for frequency-domain analysis.

For raw image data, the absolute value of the Hilbert transform for each backscattered RF signal was calculated to obtain the envelope image. An ultrasound B-mode image was formed using a log-compressed envelope image at a dynamic range of 60 dB. In this study, the window-to-window computational scheme was selected because it supports the acquisition of local ultrasound signals with finite lengths for frequency-domain analysis. The algorithm is illustrated in Fig. 3, and the details are provided as follows^24^.

First, the envelope image *R*_*k*_ at each time point (*k* = 0, 1, 2, …, *n*; *R*_0_ represents the reference data) can be expressed as

1 $${R}_{k}(x,y)=[\begin{array}{ccccc}{r}_{k(1,1)} & {r}_{k(1,2)} & . & . & {r}_{k(1,x)}\\ {r}_{k(2,1)} & . & . & . & .\\ . & . & . & . & .\\ . & . & . & . & .\\ {r}_{k(y,1)} & . & . & . & {r}_{k(x,y)}\end{array}].$$

To acquire local envelopes ${\hat{R}}_{{\rm{k}}}$, a window (i.e., subimage) with a size of $l\times m$ is set within *R*_*k*_:

2 $${\hat{R}}_{k}=[\begin{array}{ccccc}{r}_{k(i,j)}\, & {r}_{k(i,j+1)} & . & . & {r}_{k(i,j+m)}\\ {r}_{k(i+1,j)} & . & . & . & .\\ . & . & . & . & .\\ . & . & . & . & .\\ {r}_{k(i+l,j)} & . & . & . & {r}_{k(i+l,j+m)}\end{array}],$$

where *i* and *j* refer to locations in the upper left corner of the window. The local spatial-domain CBE value (denoted by ${\hat{\eta }}_{s}$) assigned as the new pixel located at the center of the window can be calculated using Eq. (3):

3 $${\hat{\eta }}_{s}=10\,{\log }_{10}\,\left(\frac{E\,[{({\hat{R}}_{k})}^{2}]}{E\,[{({\hat{R}}_{0})}^{2}]}\right),$$

where *E*[∙] represents the statistical mean. The window then moves throughout *R*_*k*_ and *R*_0_ according to a window overlap ratio (WOR) for generating a CBE map. A spatial-domain CBE image with the same size as the original image can be obtained after data interpolation^24^:

4 $${\eta }_{s}(x,y)=[\begin{array}{ccccc}{\hat{\eta }}_{s(1,1)} & {\hat{\eta }}_{s(1,2)} & . & . & {\hat{\eta }}_{s(1,x)}\\ {\hat{\eta }}_{s(2,1)} & . & . & . & .\\ . & . & . & . & .\\ . & . & . & . & .\\ {\hat{\eta }}_{s(y,1)} & . & . & . & {\hat{\eta }}_{s(x,y)}\end{array}].$$

In this study, the WOR was set at 50% to compromise CBE image resolution and computational complexity, and the side length corresponding to three times the pulse length of the ultrasound transducer (6.9 mm) was used to determine the dimension of the sliding window^24^.

For frequency-domain CBE imaging, the algorithmic scheme is nearly the same as that of the conventional approach in the spatial domain. The major difference is that the envelope image data serving as the input for the algorithm are replaced by the raw data of the backscattered RF signals for sliding window processing and Fourier transform computations. For each local RF signal acquired by the sliding window, the Fourier transform is performed and squared to obtain the power spectrum, which is used to establish the maximum magnitude in a gated frequency range between 2.5 and 3.5 MHz. Such a frequency range does not overlap with that of the HIFU probe, reducing the HIFU signal’s effects on the CBE estimation. Thus, in the frequency domain, the local CBE value, denoted by ${\hat{\eta }}_{f}$, is estimated as follows:

5 $${\hat{\eta }}_{f}=10\,{\log }_{10}\,\left(\frac{\max \,[|{\rm{FT}}({\hat{R}}_{k}){|}^{2}]}{\max \,[|{\rm{FT}}({\hat{R}}_{0}){|}^{2}]}\right),$$

where max[∙] represents the maximum value in the predetermined bandwidth, and FT represents the Fourier transform. Using the sliding window technique for the spatial-domain CBE imaging, the frequency-domain CBE image can be obtained as

6 $${\eta }_{f}(x,y)=[\begin{array}{ccccc}{\hat{\eta }}_{f(1,1)} & {\hat{\eta }}_{f(1,x)} & . & . & {\hat{\eta }}_{f(1,x)}\\ {\hat{\eta }}_{f(2,1)} & . & . & . & .\\ . & . & . & . & .\\ . & . & . & . & .\\ {\hat{\eta }}_{f(y,1)} & . & . & . & {\hat{\eta }}_{f(x,y)}\end{array}].$$

### Data analysis

The temperature changes, which were measured using the thermocouple, as functions of time under HIFU exposures using different output powers were plotted. According to the previous study^24^, changes in the magnitude of the CBE during heating were evaluated by taking the absolute values of the spatial- and frequency-domain CBE images (denoted by |*η*_*s*_| and |*η*_*f*_|, respectively). For each |*η*_*s*_| and |*η*_*f*_| image, a square region of interest (ROI) measuring 2 mm × 2 mm was placed on the focal spot to calculate the average of the CBE magnitude (denoted by ${\overline{|{\eta }_{s}|}}_{focalspot}$ and ${\overline{|{\eta }_{f}|}}_{focalspot}$, respectively) for temperature change comparisons. An ROI of the same size was also placed at a location 2 cm from the right side of the focal spot for calculating the CBE values of unheated regions (denoted by ${\overline{|{\eta }_{s}|}}_{background}$ and ${\overline{|{\eta }_{f}|}}_{background}$, respectively). A contrast factor was then calculated to evaluate the contrast of CBE imaging in monitoring the focal spot, as defined by

7 $${\rm{CF}}=20\,{\log }_{10}\left(\frac{{\overline{|\eta |}}_{focalspot}}{{\overline{|\eta |}}_{background}}\right).$$

The changes in the contrast factor with time obtained from |*η*_*s*_| and |*η*_*f*_| images were plotted. The Pearson correlation coefficient *r* was calculated to evaluate the correlation between CBE values and temperature. An independent *t*-test was used to calculate the probability value *p* identifying significant differences in contrast between spatial- and frequency-domain CBE images (*p* < 0.05 was considered statistically significant).

## Results

**Figure 4:**
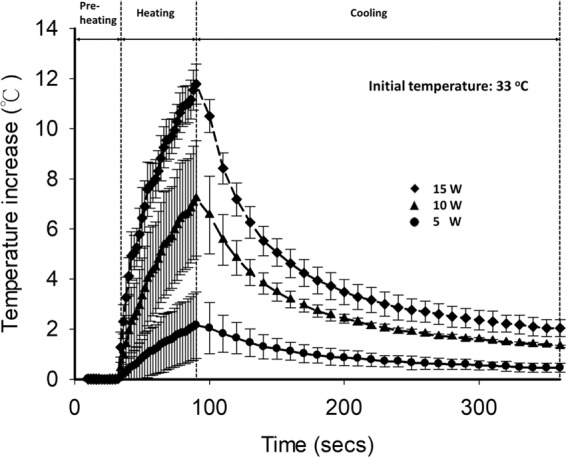
Temperature change measured close to the HIFU focal spot as a function of time for each output power. During HIFU heating, the temperatures increased by 2.2 ± 1.2 °C, 7.2 ± 2.1 °C, and 11.8 ± 0.7 °C when the HIFU output power was 5, 10, and 15 W, respectively.

**Figure 5:**
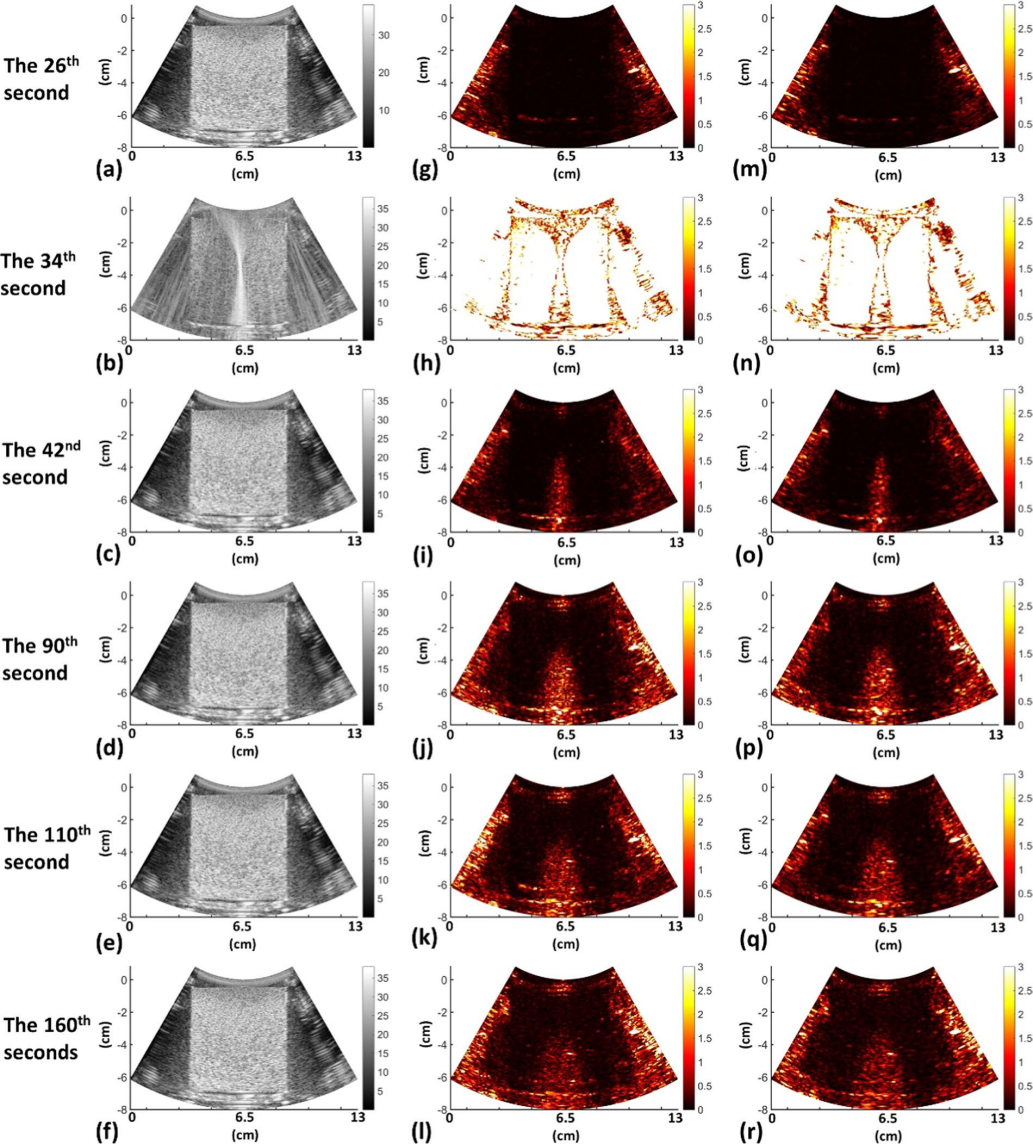
Typical ultrasound B-mode, spatial-, and frequency-domain CBE images obtained at different time points when 15-W HIFU was used. (**a**–**f**) B-mode; (**g**–**l**) spatial-domain; and (**m**–**r**) frequency-domain CBE images. Frequency-domain CBE imaging inherited the ability of the conventional spatial-domain approach in ultrasound thermal imaging.

Figure 4 demonstrates the temperature change measured close to the HIFU focal spot as a function of time for each output power. During the period of HIFU heating, the temperature increased by 2.2 ± 1.2 °C, 7.2 ± 2.1 °C, and 11.8 ± 0.7 °C when exposed to HIFU of 5, 10, and 15 W, respectively. The temperature started to decrease after HIFU heating was turned off and gradually returned to the initial temperature. Considering that the temperature variation during HIFU exposure is relatively limited, the thermocouple artifacts related to viscous heating could be ignored (also not observed in the images in Fig. 5).

Figure 5 depicts the typical ultrasound B-mode and CBE (|*η*_*s*_| and |*η*_*f*_|) images obtained at various stages of HIFU exposure. No CBE information was detected in the images prior to HIFU heating, as shown in the images at the 26^th^ s. The images at the 34^th^ s referred to those obtained under the exposure of the HIFU beam and exhibited significant acoustic interference artifacts. During HIFU heating, the brightness of the |*η*_*s*_| and |*η*_*f*_| images corresponding to the focal spot increased, signifying that spatial- and frequency-domain CBE images can visualize temperature changes, as shown in the images at the 42^nd^ and 90^th^s (the image data were collected in the HIFU-off intervals). However, tail-shaped artifacts were observed behind the HIFU focal spot with the two types of CBE imaging approaches. When HIFU heating was off, the overall brightness of the CBE images decreased, indicating that |*η*_*s*_| and |*η*_*f*_| images can also depict a decrease in temperature during the cooling period, as shown in the images at the 110^th^ and 160^th^s.

**Figure 6:**
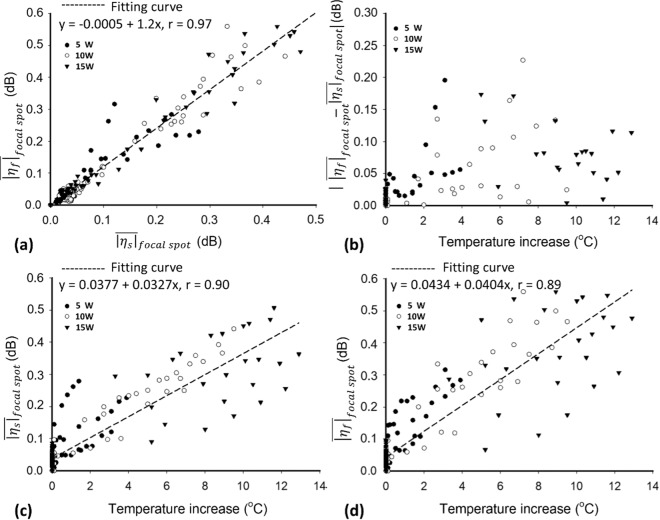
A total of the experimental data obtained from 5, 10, and 15 W were used for comparison analysis. (**a**) Correlation between ${\overline{|{\eta }_{s}|}}_{focalspot}$ and ${\overline{|{\eta }_{f}|}}_{focalspot}$ values. The frequency- and spatial-domain CBE values were strongly correlated (*r* = 0.97). (**b**) Difference between the ${\overline{|{\eta }_{s}|}}_{focalspot}$ and ${\overline{|{\eta }_{f}|}}_{focalspot}$ values increased as the temperature change increased. (**c**) Relationship between the ${\overline{|{\eta }_{s}|}}_{focalspot}$ value and temperature change (*r* = 0.90). (**d**) Relationship between the ${\overline{|{\eta }_{f}|}}_{focalspot}$ value and temperature change (*r* = 0.89).

Moreover, further analysis indicated that the frequency- and spatial-domain CBE values were closely correlated (*r* = 0.97), as shown in Fig. 6(a). For each power, the increase in temperature did not result in a significant monotonous change in the difference between ${\overline{|{\eta }_{s}|}}_{focalspot}$ and ${\overline{|{\eta }_{f}|}}_{focalspot}$ (Fig. 6(b)); in particular, the ${\overline{|{\eta }_{f}|}}_{focalspot}$ value demonstrated a similar performance to the ${\overline{|{\eta }_{s}|}}_{focalspot}$ value in describing temperature changes (*r* = 0.89), as supported by the results in Fig. 6(c,d). The aforementioned findings reveal that frequency-domain CBE imaging inherits the ability of the conventional spatial-domain approach in ultrasound thermal imaging.

**Figure 7:**
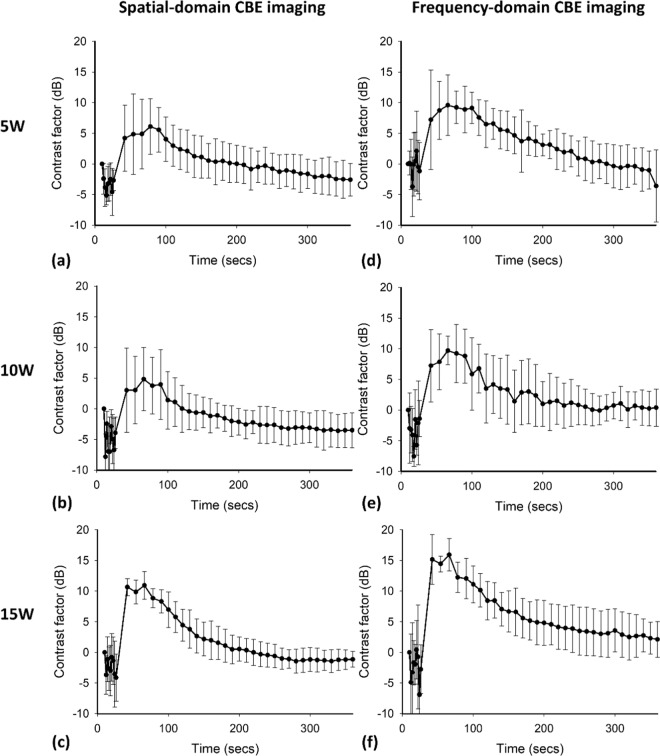
Contrast factors as functions of time obtained at different HIFU powers for (**a**–**c**) spatial-domain and (**d**–**f**) frequency-domain CBE imaging. The |*η*_*f*_| image exhibited higher contrast factor values in the period of HIFU heating than the conventional |*η*_*s*_| image did.

**Figure 8:**
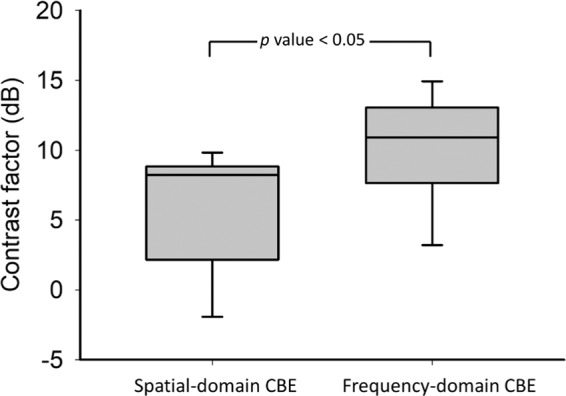
Box plots of contrast factors corresponding to spatial- and frequency-domain CBE images. Group data comparison of the contrast factor between |*η*_*s*_| and |*η*_*f*_| imaging indicated *p* < 0.05.

To compare the performance of spatial- and frequency-domain CBE imaging in HIFU monitoring, the contrast factors of the |*η*_*s*_| and |*η*_*f*_| images as functions of time obtained at various output powers are depicted in Fig. 7. The |*η*_*f*_| image exhibited higher contrast factor values during the HIFU heating period than the conventional |*η*_*s*_| image did, signifying that frequency-domain CBE imaging yielded improved performance in visual localization of the HIFU focal spot. In particular, the group data comparison of the contrast factor between |*η*_*s*_| and |*η*_*f*_| imaging indicated that the image contrast obtained using the frequency-domain technique increased by 2.5-fold (4 dB) compared with that obtained using conventional CBE imaging (*p* < 0.05), as shown in Fig. 8.

## Discussion

### Significance of this study

This study is the first to go beyond the spatial domain and develop an ultrasound CBE imaging method from the perspective of the frequency domain for reducing the effects of noises on conventional CBE imaging during HIFU treatment. The feasibility of the proposed method in HIFU focal spot localization was demonstrated through phantom experiments. The results showed that, compared with conventional spatial-domain CBE imaging, frequency-domain CBE imaging provided improved image contrast for HIFU monitoring; the CBE value also maintained a proportional relationship with temperature change.

### Mechanisms of the CBE relationship with temperature

The physical mechanisms underlying the relationship between the CBE and temperature can be explained using two models proposed previously. According to the first model, the scatterers in biological soft tissues can be acoustically divided into lipid- and aqueous-based microstructures. With increasing temperature, lipid and aqueous scatterers contribute increasing and decreasing CBE values, respectively^11–13^. As mentioned in the Introduction, this is due to the thermal effects exerted on the backscatter coefficients of scatterers. The second model was proposed to describe the mechanism for the temperature response of the CBE^27^. The increase in temperature during heating alters the speed of sound, affecting the formation of backscattered waveforms resulted from wave interference. Under this condition, local changes in speckle patterns cause changes in CBE. Based on the current experimental design, the behaviors of the CBE during HIFU procedure can be explained by using the above two models that were established at temperatures between 37 and 50 °C. Currently, a more general CBE model that can support interpretations of the CBE for a wider temperature range is still not available. However, the effects of gas bubble formation, tissue necrosis, and stiffness increase will be dominant factors that need to be considered for high-temperature HIFU exposures.

### Tail artifact in CBE imaging

In ultrasound thermal detection, reduced quality of ultrasound temperature imaging due to artifacts observed behind heated regions is an unavoidable problem. For example, when the echo time shift is used for imaging temperature change, ripple patterns typically appear under the heated region and severely corrupt temperature estimates at deeper locations^9^. In brief, the thermo-acoustic lens effect due to local changes in the sound speed (caused by temperature changes), like phase aberrations for the imaging system, results in a ripple. Therefore, smoothing along both axial and lateral directions has been recommended for the reduction of ripple artifacts^9^. Similar issues (tail-shaped artifacts) also affect ultrasound CBE imaging, regardless of whether spatial- or frequency-domain approaches are used, as per the current findings. However, tail artifacts in CBE images are not caused by the thermal lens effect; instead, they are caused by both refraction of ultrasound and changes in acoustic attenuation^23^. As suggested in a previous report^23^, tail artifacts can be partially suppressed by image fusion of both CBE and echo-time-shift mapping. Under this condition, replacing a conventional CBE approach with frequency-domain CBE imaging may further improve tail artifact suppression through the image fusion technique, because frequency-domain CBE imaging provides an improved contrast compared with conventional CBE imaging, as supported by the findings in this study.

### Limitations and future work

Monitoring higher power HIFU treatment (such as HIFU ablation) was not considered; therefore, the use of frequency-domain CBE imaging for monitoring high-temperature changes is yet to be explored. HIFU heating ranging from 36 to 43 °C was shown to activate drugs encapsulated by thermally sensitive liposomes^21^; therefore, the role of frequency-domain CBE imaging in HIFU-assisted drug delivery and release is worth exploring. Moreover, the initial temperature in each experiment was not controlled at a physiological temperature due to the limitation of the specification for the temperature controller. Such a condition may result in some unpredictable biases in estimating CBE values. However, this does not affect the primary conclusions on the advantages of frequency-domain CBE imaging. In addition, we believe that the suppression of artifacts in CBE imaging is a worthwhile and valuable development direction, but so far little research has been devoted to this topic, especially to solving the problem in the frequency domain. Finally, further investigation is merited using animal models to obtain additional validation. In particular, the effect of necrosis may occur and needs to be considered in practical applications to biological tissues. Tissue necrosis may result in acoustic attenuation, which further causes frequency downshift and changes in the amplitude distribution of the spectrum^28^. In this circumstance, the uncertainty may exist in the estimation of frequency-domain CBE if we include all the signal components in a specific frequency range for the analysis. Therefore, we used Eq. (5) as the definition of the frequency-domain CBE in this preliminary study. However, the algorithm can be flexibly modified according to future needs in practice (e.g., considerations of attenuation compensation).

## Conclusions

This study proposed a new method for ultrasound CBE imaging based on frequency-domain analysis. Phantom experiments were conducted to validate the performance of the proposed frequency-domain CBE imaging in HIFU focal spot localization. The results showed that frequency-domain CBE imaging inherits the advantage of conventional spatial-domain CBE imaging in ultrasound thermal monitoring. However, the superiority of the frequency-domain approach over the conventional method was that the CBE map generated provided an improved contrast to visually monitor the HIFU focal spot. In the future, the technique of image construction in the frequency domain may be a new strategy for contrast enhancement in CBE imaging for focal spot localization in HIFU treatment planning. Tail-artifact-related problems should be resolved prior to subsequent practical applications.
